# Risk Stratification for Hepatitis B Virus Reactivation in Kidney Transplant Recipients With Resolved HBV Infection

**DOI:** 10.3389/ti.2023.11122

**Published:** 2023-04-13

**Authors:** Hsin-Ju Tsai, Ming-Ju Wu, Cheng-Hsu Chen, Sheng-Shun Yang, Yi-Hsiang Huang, Yan-Zin Chang, Horng-Rong Chang, Teng-Yu Lee

**Affiliations:** ^1^ Institute of Medicine, Chung Shan Medical University, Taichung, Taiwan; ^2^ Division of Gastroenterology and Hepatology, Department of Internal Medicine, Taichung Veterans General Hospital, Taichung, Taiwan; ^3^ Department of Post-Baccalaureate Medicine, College of Medicine, National Chung Hsing University, Taichung, Taiwan; ^4^ Division of Nephrology, Department of Internal Medicine, Taichung Veterans General Hospital, Taichung, Taiwan; ^5^ School of Medicine, Chung Shan Medical University, Taichung, Taiwan; ^6^ Ph.D. Program in Translational Medicine, National Chung Hsing University, Taichung, Taiwan; ^7^ Institute of Biomedical Sciences, National Chung Hsing University, Taichung, Taiwan; ^8^ Department of Life Science, Tunghai University, Taichung, Taiwan; ^9^ Division of Gastroenterology and Hepatology, Department of Medicine, Taipei Veterans General Hospital, Taipei, Taiwan; ^10^ Institute of Clinical Medicine, National Yang Ming Chiao Tung University, Taipei, Taiwan; ^11^ Department of Clinical Laboratory, Drug Testing Center, Chung Shan Medical University Hospital, Taichung, Taiwan; ^12^ Division of Nephrology, Department of Internal Medicine, Chung Shan Medical University Hospital, Taichung, Taiwan

**Keywords:** immunosuppression, renal transplantation, hepatitis B, reversion, antiviral therapy

## Abstract

The prophylaxis strategy for hepatitis B virus (HBV) reactivation in kidney transplant recipients (KTRs) with resolved HBV infection remains unclear. In this hospital-based retrospective cohort study, consecutive KTRs with resolved HBV infection were screened from the years 2000 through 2020. After excluding confounding conditions, 212 and 45 patients were respectively recruited into Anti-HBs positive and Anti-HBs negative groups. Cumulative incidences of, and subdistribution hazard ratios (SHRs) for HBV reactivation were analyzed after adjusting the competing risk. During a median 8.3 (mean 8.4 ± 4.9) years of follow-up, the 10-year cumulative incidence of HBV reactivation was significantly higher in Anti-HBs negative group when compared to that in Anti-HBs positive group (15.2%, 95% CI: 3.6–26.7 vs. 1.3%, 95% CI: 0.0–3.0; *p* < 0.001). In multivariable regression analysis, absence of anti-HBs (SHR 14.2, 95% CI: 3.09–65.2; *p* < 0.001) and use of high-dose steroids, i.e., steroid dose ≥20 mg/day of prednisolone equivalent over 4 weeks (SHR 8.96, 95% CI: 1.05–76.2; *p* = 0.045) were independent risk factors related to HBV reactivation. Accordingly, the 10-year cumulative incidence of HBV reactivation occurring in patients with two, one and zero risk factors was 42.7% (95% CI: 0.0–87.1), 7.9% (95% CI: 1.2–14.7) and 0%, respectively (*p* < 0.001). In conclusion, the strategy of HBV antiviral prophylaxis may be defined according to the risk stratification.

## Graphical Abstract

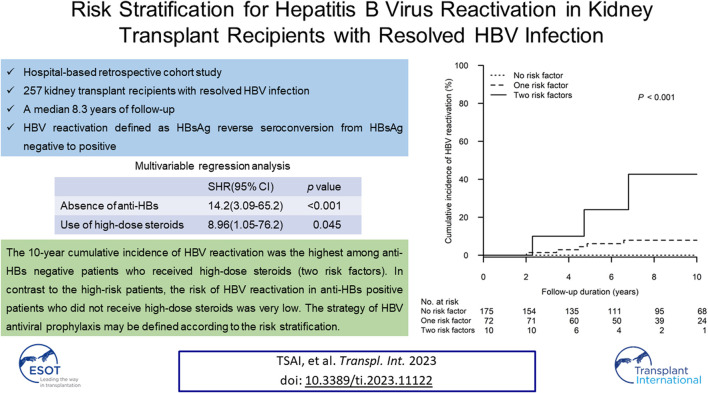

## Introduction

Kidney diseases are the leading cause of solid organ transplantation globally, with more than 100,000 patients receiving a kidney transplant per year (1). Although hepatitis B virus (HBV) infection may not directly involve the pathogenesis of kidney diseases, hepatitis B progression can be the major cause of either patient morbidity or mortality after kidney transplantation (2). In kidney transplant patients with chronic HBV infection, immunosuppressive therapy can result in rapid liver fibrosis progression, and patients may in turn die of liver-related complications (2, 3). In patients with resolved HBV infection, i.e., those with positive antibody to hepatitis B core antigen (anti-HBc) but negative hepatitis B surface antigen (HBsAg) in the blood, although the risk of hepatitis B progression is much lower than that in HBsAg-positive patients, HBV may still exist somewhere in the body; e.g., in the nucleus of hepatic cells (4). While the host immune system is suppressed, HBV replication may be reactivated, i.e., the reappearance of HBsAg and HBV deoxyribonucleic acid (DNA) in blood (5). Previous studies have reported that immunosuppressive chemotherapy could induce both severe hepatitis B flare and death in patients with resolved HBV infection (6–8), where nucleos(t)ide analogue (NA) therapy can be considered for patients in the high-risk stratification.

With a high risk of HBV reactivation and liver-related mortality in kidney transplant recipients (KTRs) with chronic HBV infection, i.e., positive HBsAg, life-long prophylactic NA therapy has been recommended in the practice guidelines (9–11). However, with a relatively lower risk of HBV reactivation, ranging from 2% to 9.6% in KTRs with resolved HBV infection (12–18), current guidelines only suggest regular follow-ups, rather than long-term NA therapy prophylaxis (10, 11). However, several clinical studies have observed that the risk of HBV reactivation may be particularly higher in patients with resolved HBV infection, but without antibody to HBsAg (anti-HBs) (6, 19). Although the absence of anti-HBs could be a risk factor for HBV reactivation in KTRs with resolved HBV infection, the role in which other risk factors may play remains largely unknown (12–18).

In previous studies of patients with resolved HBV infection, immunosuppressants could be seen as being strongly related to HBV reactivation (7, 20), however their role in KTRs has not yet been systemically investigated. For example, corticosteroids are commonly used as the backbone of immunosuppression therapy, with a dose ranging from an ultra-high dose of pulse therapy or a high dose of rejection therapy, to low-dose maintenance therapy (21); however, the association between steroid dosages and the risk of HBV reactivation remains unclear. For patients at a high risk of HBV reactivation, severe liver complications may be avoided or prevented. We therefore aimed to conduct a long-term cohort study to assess the timing and severity of HBV reactivation in KTRs with resolved HBV infection, as well as comprehensively analyze any possible risk factors which may be of concern.

## Patients and Methods

### Study Design

This retrospective cohort study was conducted at Taichung Veterans General Hospital (VGHTC), a tertiary medical center in central Taiwan. Any end-stage renal disease patient who had received kidney transplantation at VGHTC between 1st January 2000 and 31st December 2020 was recruited. The study subjects were followed up for clinical outcomes until 31st December of 2021. The medical records of the study subjects were retrieved for analysis. This study was approved by the Institutional Review Board of VGHTC (CE21059B).

### Study Subjects

**FIGURE 1:**
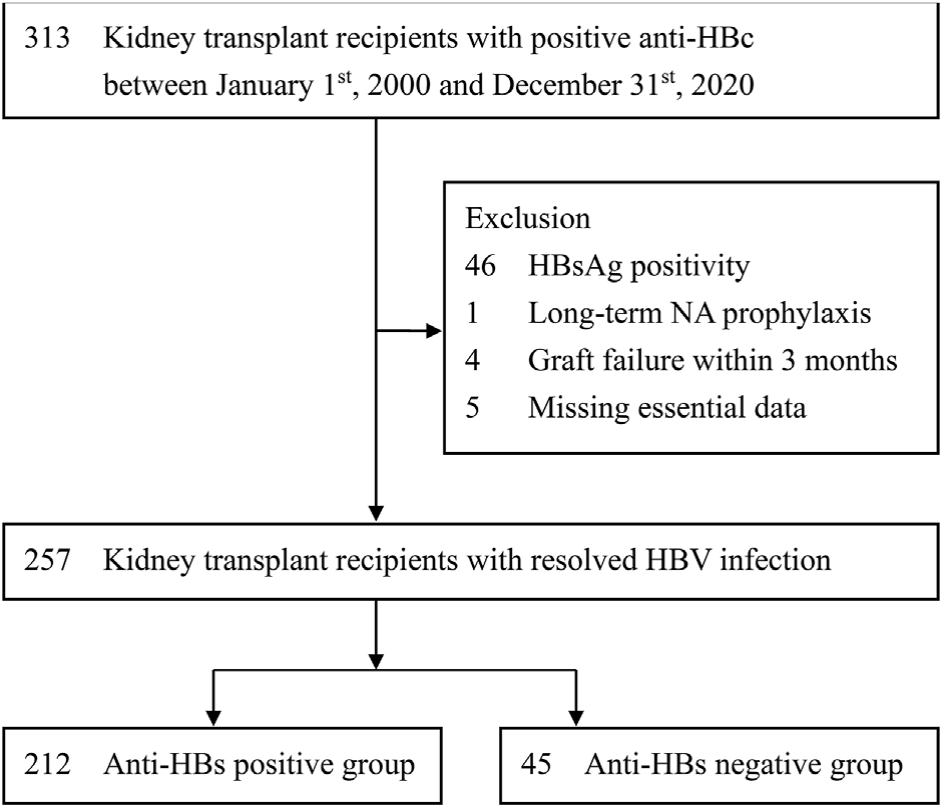
Selection of study subjects. Anti-HBc, antibody to hepatitis B core antigen; anti-HBs, antibody to HBsAg; HBV, hepatitis B virus; HBsAg, hepatitis B surface antigen; NA, nucleos(t)ide analogue.

The patient selection process is shown in Figure 1. The inclusion criteria were as follows: 1) KTRs and 2) positive anti-HBc. The exclusion criteria were as follows: 1) positive HBsAg, 2) receiving long-term prophylactic NA therapy, 3) kidney graft failure within 3 months after transplantation, and 4) incomplete essential data. The study subjects were followed up for 10 years or until the dates of 1) HBV reactivation, 2) kidney graft failure, 3) patient mortality, 4) receiving of chemotherapy for a newly diagnosed malignancy, 5) loss follow-up, or 6) 31st December of 2020. According to the positivity of serum anti-HBs before kidney transplantation, patients were recruited into anti-HBs positive or anti-HBs negative group.

### HBV Reactivation and Hepatitis Flare

The primary endpoint was HBV reactivation, which was defined as HBsAg reverse seroconversion from HBsAg-negative to HBsAg-positive (10). According to the clinical practice routines in our hospital, periodical surveillance for HBV reactivation was performed after kidney transplantation, i.e., serum ALT every 3 months and serum HBsAg every 6–12 months. In addition, serum HBsAg and HBV DNA would be additionally checked if serum ALT was increased for at least twice the baseline level or above the upper limit of normal (ULN). The secondary endpoints included HBV-associated hepatitis in combination with HBV reactivation and hepatitis flare. Hepatitis flare is defined as alanine aminotransferase (ALT) increase >3 times baseline and >100 U/L (10). Other endpoint, including severe flare, was defined as hepatitis B flare (HBV DNA level >2,000 IU/mL and ALT > 5x the ULN) with jaundice (total bilirubin ≥2 mg/dL), and/or coagulopathy (prothrombin time prolongation ≥3 s) (22). The ULN of ALT was defined according to the updated American Association for the Study of Liver Diseases criteria (>25 U/L for females and >35 U/L for males) (10).

### Risk Factors Assessment

The data including blood type ABO incompatibility and human leukocyte antigens (HLA) mismatch numbers were collected. Hepatitis C virus (HCV) co-infection in patients was defined as those who were hepatitis C antibody positive with a detected HCV viral load in their serum. We retrieved the immunosuppressants used during induction (rituximb, basiliximab, thymoglobulin and others), as well as the standard triple agents in maintenance (calcineurin inhibitor, mycophenolate mofetil and corticosteroids). The calcineurin inhibitors included cyclosporine and tacrolimus. The data on sirolimus or everolimus combination with the triple agents used in maintenance was also captured. Steroid therapy is a part of immunosuppressive regimens used for induction, maintenance and anti-rejection therapy. Detailed information regarding steroid therapy, including dosage and duration, was comprehensively obtained from medical records. We converted dosages of various steroid therapies into equivalent doses of prednisolone based on anti-inflammatory potency (23). The average steroid dose was defined as the total amount of steroid dosage used in maintenance divided by the sum of the days of steroid treatment. Peak steroid dose was defined as the maximal steroid dosage which persisted at least 4 weeks in maintenance. We set up three strata of peak steroid dose using prednisolone equivalents as rates of <10 mg/day, 10–19 mg/day and ≥20 mg/day (24). After kidney transplantation, allograft rejection development would be suspected as patients experienced a rising serum creatinine or worsening proteinuria. Acute rejection was defined by the presence of pathologic evidence seen on a kidney allograft biopsy (21). The data on rejection episodes and treatments were collected.

### Statistical Analyses

Continuous variables were expressed in median with interquartile ranges (IQRs), while categorical variables were presented as both number and percentage. Continuous variables were compared by the Mann-Whitney U test, while categorical variables were compared through use of either the Chi-square test or Fisher’s exact test. Cumulative incidence rates of HBV reactivation or hepatitis flare were calculated and compared by using a Fine-Gray method and Kaplan-Meier method, respectively (25). The differences in the full time-to-event distributions among the study groups were compared by a log-rank test. Renal graft failure or patient mortality before HBV reactivation was treated as a competing event. We further performed univariable analysis to identify any potential risk factors for HBV reactivation, with independent risk factors being determined according to the results of multivariable regression analysis. Subdistribution hazard ratios (SHRs) were obtained in Cox proportional hazard models and adjusted on the basis of the subdistribution of the competing risk. The R-package “cmprsk” was used for the purpose of competing risks regression (26). A two-tailed *p* < 0.05 was considered statistically significant. We managed the data using SAS 9.3 software (SAS Institute, Inc., Cary, NC, USA).

## Results

### Study Subjects

As shown in Figure 1, after excluding those with confounding conditions, 257 patients were identified for final analysis. According to the positivity of serum anti-HBs, 212 and 45 patients were respectively recruited into anti-HBs positive and anti-HBs negative groups. As shown in Table 1, apart from age, nearly all the baseline patient characteristics do not reveal significant differences between the two study groups. The median age was younger in the anti-HBs positive group than that in the anti-HBs negative group (49.0 vs. 51.5 years). The proportions of other possible risk factors were not significantly different between the two study groups, including gender, HCV co-infection, HBsAg-positive donor, blood type ABO incompatible transplant, HLA mismatch, immunosuppressive regimens, short-term NA prophylaxis during induction, episodes of biopsy proven acute rejection, and treatment for acute rejection. Moreover, we also analyzed the details surrounding steroid use, including average steroid dose and peak steroid dose, which were also similar in the two study groups. The median follow-up duration was 8.3 (IQR, 4.4–11.9) years, with a mean duration of 8.4 ± 4.9 years (Supplementary Figure S1). The median follow-up duration was not significantly different in the two study groups. (anti-HBs positive vs. anti-HBs negative: 8.8 [IQR: 4.3–10.0] vs. 6.8 [IQR, 4.5–8.6] years; *p* = 0.084).

**TABLE 1:** Baseline characteristics of study subjects.

	Positive anti-HBs *n* = 212	Negative anti-HBs *n* = 45	*p*-value
Age, years	49.0 (39.5–54.0)	51.5 (47.9–60.5)	0.008
Male, n (%)	112 (52.8)	26 (57.8)	0.660
HCV co-infection, n (%)	16 (7.5)	5 (11.1)	0.622
HBsAg-positive donor, n (%)	23 (10.8)	3 (6.7)	0.587
Positive Anti-HBs donor, n (%)	169 (79.7)	33 (73.3)	0.343
Donor source, n (%)			0.303
Living donor	96 (45.3)	16 (35.6)	
Deceased donor	116 (54.7)	29 (64.6)	
Prior history of renal transplant, n (%)	3 (1.4)	3 (6.7)	0.068
ABO-incompatibility, n (%)	30 (17.9)	7 (15.6)	0.870
HLA mismatch numbers	2 (0.3–3.0)	2 (0.0–3.0)	0.144
Induction therapy, n (%)			0.772
No	51 (26.0)	13 (29.5)	
Rituximab	11 (5.6)	1 (2.3)	
Basiliximab	107 (54.6)	23 (52.3)	
Thymoglobulin	27 (13.8)	7 (15.9)	
NA prophylaxis during induction, n (%)	16 (7.5)	5 (11.1)	0.622
Duration of NA prophylaxis, months	4.2 (0.9–6.5)	5.6 (0.9–7.1)	0.934
Maintenance immunosuppressants, n (%)^a^			0.886
Cyclosporine + MMF + steroids	30 (14.2)	6 (13.3)	
Tacrolimus + MMF + steroids	182 (85.8)	39 (86.7)	
Maintenance steroid^b^			
Average dose, mg/day	6.0 (5.2–7.5)	6.1 (5.1–8.3)	0.545
Peak dose^c^			0.748
<10 mg/day	77 (36.3)	15 (33.3)	
10–19 mg/day	98 (46.2)	20 (44.4)	
≥20 mg/day	37 (17.5)	10 (22.2)	
Sirolimus or everolimus combination, n (%)	96 (45.3)	13 (28.9)	0.064
Acute rejection episodes, n (%)			0.979
No	138 (65.1)	30 (66.7)	
Once	39 (18.4)	8 (17.8)	
≥2 episodes	35 (16.5)	7 (15.6)	
Treatment for acute rejection, n (%)			0.712
Rituximab	12 (16.2)	3 (20.0)	
Methylprednisolone pulse therapy	62 (83.8)	12 (80.0)	

^a^ Major immunosuppressants used in maintenance.

^b^ Values represent prednisolone equivalents.

^c^ Peak dose defined as the maximal steroid dosage which persisted ≥4 weeks in maintenance.

Continuous variables are expressed in median (interquartile range).

Anti-HBs, antibody to HBsAg; HBsAg, hepatitis B surface antigen; HCV, hepatitis C virus; HLA, human leucocyte antigen; MMF, mycophenolate mofetil; NA, nucleos(t)ide analogue.

### HBV Reactivation and Hepatitis Flare

**FIGURE 2:**
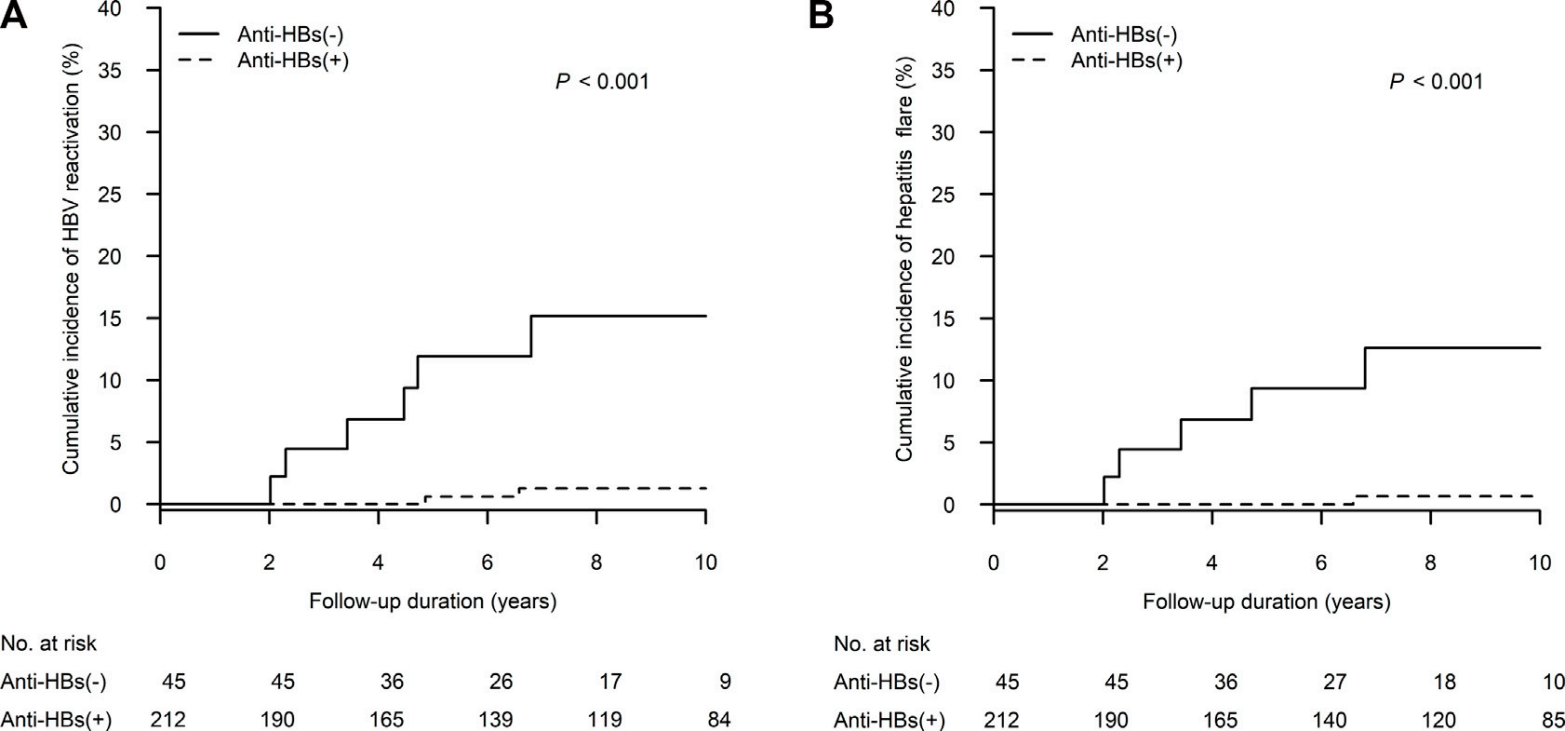
The cumulative incidence of **(A)** HBV reactivation and **(B)** hepatitis flare was higher in patients without anti-HBs than in patients with anti-HBs in competing risks regression. Anti-HBs, antibody to hepatitis B surface antigen; HBV, hepatitis B virus.

As shown in Figure 2A, the 10-year cumulative incidence of HBV reactivation was significantly higher in the anti-HBs negative group when compared to that in the anti-HBs positive group (15.2%, 95% confidence interval [CI]: 3.6%–26.7% vs. 1.3%, 95% CI: 0.0–3.0; *p* < 0.001). Table 2 presents the details of patients experiencing HBV reactivation: six in the anti-HBs negative group and two in the anti-HBs positive group. HBV reactivation happened during the period of 2–6 years after kidney transplant, and often appeared within 1 year after tapering steroid administration from its peak dose. Reappearance of HBsAg also combined with HBV DNA level >2,000 IU/mL and ALT elevation > 2x ULN in all of these patients. Moreover, five anti-HBs negative patients and one anti-HBs positive patient experienced hepatitis flare, which is defined as ALT increase >3 times baseline and >100 U/L (10). Eight patients who developed HBV reactivation were all hepatitis B e antigen (HBeAg) negative at baseline, and three with antibody to HBeAg. Four (4/8; 50%) patients became HBeAg positive during HBV reactivation.

**TABLE 2:** Characteristics of the patients with HBV reactivation.

No.	Age (years)	Gender	Anti-HBs	Maintenance steroid^a^		Time to HBV reactivation		Data during HBV reactivation					NA therapy	HBsAg loss after NA therapy
				Avg. dose (mg/day)	Peak dose^b^ (mg/day)	From transplant (months)	From peak steroid tapering (months)	Steroid dose^a^ (mg/day)	HBV DNA (log IU/mL)	Bilirubin^c^ (mg/dL)	ALT^c^ (U/L)	HbeAg presence		
1	50	F	Neg.	5.6	5	41	5	5	6.20	1.1	118	Neg	LAM	Yes
2	39	M	Neg.	9.1	10	54	12	5	8.04	1.0	61	Pos.	ETV	No
3	59	M	Neg.	5.7	10	24	2	5	3.96	0.8	158	Neg	LAM	Yes
4	48	F	Pos.	7.1	20	79	12	5	7.95	1.7	151	Pos.	ETV	Yes
5	33	F	Pos.	7.9	30	58	1	20	5.96	0.4	52	Pos.	ETV	No
6	51	F	Neg.	7.2	30	82	10	5	6.87	1.1	812	Pos.	ETV	No
7	60	F	Neg.	10.4	20	28	14	5	8.23	0.6	119	Neg	ETV	No
8	48	F	Neg.	7.0	40	57	8	10	6.20	7.9	326	Neg	ETV	No

^a^ Values represent prednisolone equivalents.

^b^ Peak dose defined as the maximal steroid dosage which persisted ≥4 weeks in maintenance.

^c^ Peak level during HBV reactivation.

ALT, alanine aminotransferase; Anti-HBs, antibody to HBsAg; DNA, deoxyribonucleic acid; ETV, entecavir; HBeAg, hepatitis B e antigen; HBsAg, hepatitis B surface antigen; HBV, hepatitis B virus; LAM, lamivudine; NA, nucleos(t)ide analogue.

Regarding HBV vaccination, among 47 patients were initially anti-HBs negative prior to kidney transplantation, 14 patients (14/47; 29.8%) received HBV vaccination: Two patients (2/14; 14.3%) produced durable anti-HBs, and they were thus sorted into the anti-HBs positive group. Only one vaccinated patient (1/14; 7.1%), who failed to produce durable anti-HBs, experienced HBV reactivation after kidney transplantation. In univariable regression analysis for all the initially anti-HBs negative patients, HBV vaccination prior to transplantation was not significantly associated with a lower risk of HBV reactivation (SHR 0.61, 95% CI: 0.07–5.28; *p* = 0.656). The efficacy of HBV vaccination in preventing HBV reactivation might not be sufficiently evaluated due to the limited case numbers in this study.

The 10-year cumulative incidence of hepatitis flare was significantly higher in the anti-HBs negative group when compared to that in the anti-HBs positive group (12.6%, 95% CI: 1.9%–23.3% vs. 0.7%, 95% CI: 0.0–2.0; *p* < 0.001) (Figure 2B). Severe hepatitis flare, i.e., jaundice and ALT > 5x ULN (1), was noted in one anti-HBs negative patient. All patients diagnosed with HBV reactivation received NA therapy within 1 month after HBsAg seroreversion. Fortunately, no patient died of hepatic failure. After NA therapy, three patients (37.5%) experienced HBsAg loss again thereafter (1.2, 4 and 8.7 years after their HBV reactivation episodes).

### The Risk Factors of HBV Reactivation

As shown in Table 3, in univariable regression analysis, a negative anti-HBs status (SHR 14.3, 95% CI: 2.97–68.8; *p <* 0.001), increased average steroid daily dose (SHR 1.13 per mg of prednisolone equivalent, 95% CI: 1.04–1.23; *p* = 0.003), and a peak steroid dose ≥20 mg/day of prednisolone equivalent (SHR 8.96, 95% CI: 1.05–76.2; *p* = 0.045) were associated with the occurrence of HBV reactivation. The peak dose was defined as the maximal steroid dosage which persisted ≥4 weeks in maintenance. Furthermore, in multivariable regression analysis (Model 1), both a negative anti-HBs status (SHR 13.3, 95% CI: 2.75–64.4; *p* = 0.001) and increased average steroid daily dose (SHR 1.12 per mg of prednisolone equivalent, 95% CI: 1.02–1.23; *p* = 0.023) were significantly associated with the development of HBV reactivation. In addition, as shown in Model 2, a negative anti-HBs status (SHR 14.2, 95% CI: 3.09–65.2; *p* < 0.001) and a peak steroid dose ≥20 mg/day of prednisolone equivalent (SHR 9.20, 95% CI: 1.06–79.8; *p* = 0.044) remained the independent risk factors for HBV reactivation.

**TABLE 3:** Subdistribution hazard ratio of risk factors for HBV reactivation in univariate and multivariate competing-risks regression.

	Univariable analysis			Multivariable Model 1			Multivariable Model 2		
	SHR	95% CI	*p*-value	SHR	95% CI	*p*-value	SHR	95% CI	*p*-value
Anti-HBs Negative vs. Positive	14.3	(2.97–68.8)	<0.001	13.3	(2.75–64.4)	0.001	14.2	(3.09–65.2)	<0.001
Age per year	1.01	(0.96–1.06)	0.709						
Male vs. Female	0.30	(0.06–1.47)	0.138						
HCV co-infection	N/A^a^	-	-						
HBsAg-positive donor	1.13	(0.14–8.89)	0.904						
Positive Anti-HBs donor	0.53	(0.11–2.65)	0.442						
Living vs. Deceased donor	0.96	(0.23–3.99)	0.961						
Prior history of renal transplant	N/A^a^	-	-						
ABO-incompatibility	0.59	(0.12–2.88)	0.514						
HLA mismatch numbers	0.67	(0.40–1.12)	0.129						
Induction therapy									
No		*ref*.							
Rituximab	N/A^a^	-	-						
Others	2.64	(0.33–21.1)	0.361						
NA prophylaxis during induction	1.96	(0.25–15.2)	0.521						
Maintenance immunosuppressants									
Cyclosporine + MMF + steroids		*ref*.							
Tacrolimus + MMF + steroids	0.60	(0.12–2.93)	0.530						
Maintenance steroid^b^									
Average dose per mg/day	1.13	(1.04–1.23)	0.003	1.12	(1.02–1.23)	0.023			
Peak dose^c^									
<10 mg/day		*ref*.						*ref.*	
10–19 mg/day	1.39	(0.13–15.3)	0.788				1.50	(0.14–16.5)	0.741
≥20 mg/day	8.96	(1.05–76.2)	0.045				9.20	(1.06–79.8)	0.044
Combined sirolimus or everolimus	0.68	(0.16–2.83)	0.596						
Acute rejection episodes									
No rejection		*ref*.							
Once	0.57	(0.07–4.49)	0.589						
≥2 episodes	0.58	(0.07–4.66)	0.609						
Treatment for acute rejection									
No rejection		*ref*.							
Rituximab	N/A^a^	-	-						
MTP pulse therapy	0.71	(0.15–3.44)	0.675						

^a^ No HBV reactivation in patients with HCV co-infection, prior history of renal transplant and administration of rituximab. The associated effects of these factors could not be evaluated in the Cox proportional hazard model for HBV reactivation.

^b^ Values represent prednisolone equivalents.

^c^ Peak dose defined as the maximal steroid dosage which persisted ≥4 weeks in maintenance.

Anti-HBs, antibody to HBsAg; HBsAg, hepatitis B surface antigen; HBV, hepatitis B virus; HCV, hepatitis C virus; HLA, human leucocyte antigen; MMF, mycophenolate mofetil; MTP, methylprednisolone; NA, nucleos(t)ide analogue; N/A, not available; SHR, subdistribution hazard ratio.

**FIGURE 3:**
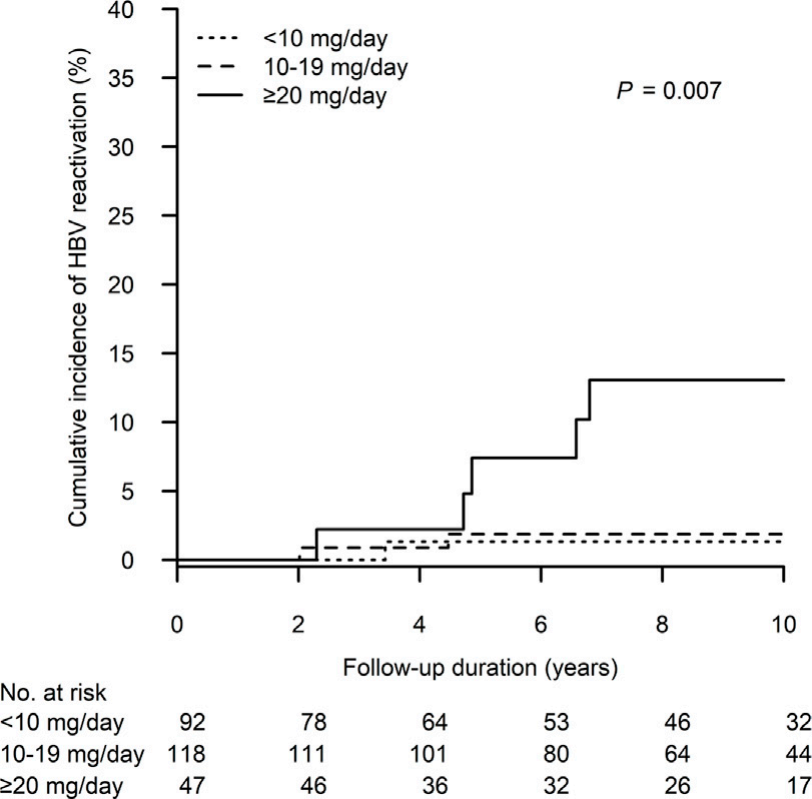
The cumulative incidence of HBV reactivation after kidney transplant according to different peak daily doses of prednisolone, or equivalent. HBV, hepatitis B virus.

**FIGURE 4:**
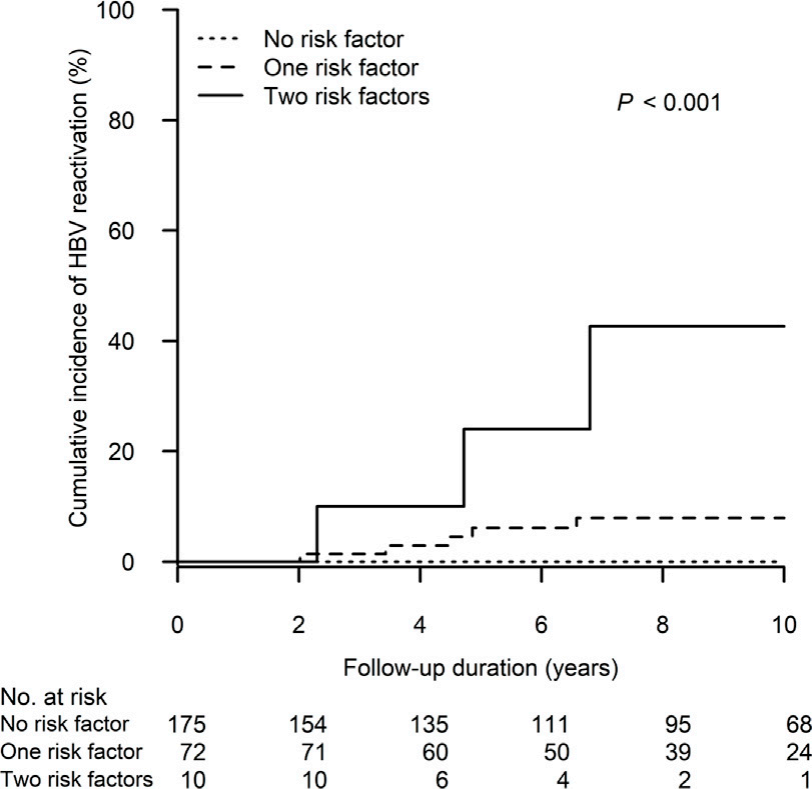
The cumulative incidence of HBV reactivation after kidney transplant in the patient groups stratified by the risk factors of HBV reactivation. The two risk factors are defined as follows (1): absence of baseline serum anti-HBs and (2) high-dose steroids, i.e., a peak steroid dose ≥20 mg/day of prednisolone equivalent which persisted ≥4 weeks in maintenance. HBV, hepatitis B virus.

Figure 3 shows the cumulative incidence of HBV reactivation in the patient groups receiving different peak steroid doses (<10, 10–19 and ≥20 mg/day of prednisolone equivalent for 4 weeks). The 10-year cumulative incidence of HBV reactivation was highest in patients who received high-dose steroids (≥20 vs. 10–19 vs. < 10 mg/day: 13.1%, 95% CI: 2.1%–24.0% vs. 1.9%, 95% CI: 0.0%–4.4% vs. 1.3%, 95% CI: 0.0–4.0, *p* = 0.007). Moreover, as demonstrated in Figure 4, we performed a risk stratification based on the independent risk factors of HBV reactivation, i.e., absence of baseline serum anti-HBs and high-dose steroids, and the 10-year cumulative incidence of HBV reactivation occurring in patients with two, one and zero risk factors was 42.7% (95% CI: 0.0–87.1), 7.9% (95% CI: 1.2–14.7) and 0%, respectively (*p* < 0.001).

## Discussion

Although HBV reactivation in 1%–10% of cases can be classified as moderate risk (24), the prophylaxis strategy for HBV reactivation in KTRs with resolved HBV infection remains unclear in the current practice guidelines. In the present study, we comprehensively collected the data on immunosuppressants and analyzed the dosages and durations of corticosteroid use. This cohort study is able to provide evidence that the absence of anti-HBs and high-dose steroid use (≥20 mg/day of prednisolone equivalent ≥4 weeks in maintenance) were both independent risk factors associated with HBV reactivation. The cumulative incidence of HBV reactivation will be the highest (>40%) among anti-HBs negative patients who received high-dose steroids, in which case antiviral therapy prophylaxis should be mandatory. In contrast to the high-risk patients, the risk of HBV reactivation in anti-HBs positive patients who did not receive high-dose steroids is very low (0%), therefore a long-term antiviral therapy prophylaxis may be waived. In these low-risk patients, a strategy involving periodic surveillance for HBV reactivation, such as HBsAg testing, may be more cost-effective than NA therapy prophylaxis. The findings of this study may provide an effective and cost-saving strategy in the use of antiviral prophylaxis, which should be valuable to both clinicians and patients.

Similar to the findings in previous studies for KTRs with resolved HBV infection, our study also demonstrates that the absence of baseline serum anti-HBs is a strong risk factor of HBsAg seroreversion after kidney transplantation (12–14). However, other risk factors may be also involved in HBV reactivation (13–15). Although the presence of anti-HBs lowered HBV reactivation risk, the risk is not totally eliminated. For patients with only one risk factor of HBV reactivation, i.e., positive anti-HBs patients who will receive high-dose steroids or negative anti-HBs patients who do not need to use high-dose steroids in this study, NA therapy prophylaxis or close monitoring for HBV reactivation should be considered. However, which strategy is more cost-effective needs further investigated. In addition, the other risk factors found in other similar studies (13–15), including age, ABO-incompatibility, rituximab use, and acute rejection, were not significantly related to HBV reactivation in this study, and their effects should be further clarified in the future studies.

To the best of our knowledge, our cohort is the first study designed to evaluate the effects of steroid therapy on the risk of HBV reactivation in KTRs with resolved HBV infection. Corticosteroids are the most widely used immunosuppressive agents, and a daily dose above 20 mg for longer than 2 weeks of prednisolone, or its equivalent, is generally considered to induce significant immunosuppression (27). HBV reactivation with active viral replication maybe occur when the host is immune suppressed (5). A systemic review suggested that steroid therapy longer than 4 weeks at a moderate (10–20 mg/day of prednisolone equivalent) or high-dose (>20 mg/day of prednisolone equivalent) may lead to HBV reactivation in 1%–10% of patients resolved HBV infection (24). In a cohort study involving rheumatic patients with resolved HBV infection, individuals experiencing HBsAg seroreversion had been exposed to a daily dose of prednisolone over 20 mg (7). Our analysis demonstrates that receiving a peak steroid dose ≥20 mg/day of prednisolone equivalent ≥4 weeks in maintenance had a major impact on risk of HBV reactivation. In addition, most HBsAg seroreversion and hepatitis flare occurred within 1 year after the tapering off of steroid administration from its peak dose. Host immune may rebound and hepatitis may develop after the withdrawal of immunosuppressants. Therefore, close surveillance of liver biochemistries, HBsAg status and HBV DNA remains essential while steroids are given in a decremental fashion. On the other hand, episodes of methylprednisolone (MTP) pulse therapy for acute rejection did not associate with HBV reactivation. Similar to our previous study for rheumatic patients with resolved HBV infection, maintained high dose oral steroid therapy, rather than short-term ultra-high dose MTP pulse therapy, increased the risk of HBV reactivation (28).

Several commonly used immunosuppressants, such as rituximab, have been evaluated for their HBV reactivation risk in previous studies, but the results remain conflicting (13–16). In studies mainly for hematologic malignancy patients receiving multi-course high-dose rituximab during chemotherapy, rituximab could lead to HBV reactivation in more than 10% of patients with resolved HBV infection (24). However, only a single-dose rituximab may be used for KTRs during induction or acute rejection. In two Japanese studies for KTRs with resolved HBV infection, rituximab was not related to an increased HBV reactivation risk (15, 16). In contrast to two Korean studies, rituximab was identified as a risk factor related to HBV reactivation, and patients might die of hepatic failure (13, 14). In the present study, the case number of rituximab users was limited (12 cases during induction and 15 cases for acute rejection), and HBV reactivation was not found during the follow-up period. However, due to the potentially fatal outcome and long-term effect reported in previous studies, careful surveillance for rituximab users remains required.

While there is insufficient evidence to recommend long-term antiviral prophylaxis for KTRs with resolved HBV infection, a limited duration of NA prophylaxis during the period of induction therapy with intensified immunosuppression may be an alternative option (10). However, the consensus regarding short-term NA prophylaxis has not been made in our hospital. Moreover, NA prophylaxis for KTRs with resolved HBV infection was not reimbursed by the National Health Insurance in Taiwan during the study period, therefore only a minority of KTRs received short-term NA prophylaxis during induction out of pocket. However, NA prophylaxis during induction was not significantly associated with HBV reactivation in our analysis.

Several limitations should be acknowledged with regards to this study. First, this is a retrospective study conducted in a transplant referral center, and some data were not completely collected, such as serum HBV DNA prior to transplantation, serum anti-HBc in donors, and the duration between resolving HBV infection and transplantation. However, the prevalence rate of occult HBV infection in patients with resolved HBV infection was low (29), and no HBV DNA was detected in our limited data. In addition, a Korean study reported that a positive anti-HBc in kidney donors was not related to HBV reactivation (14). With a high prevalence of anti-HBs positivity in Taiwanese donors, we believe that the effect of anti-HBc in donors should be insignificant in our study. A well-designed prospective study should be helpful to address the effects of these factors. Second, the incidence of HBsAg seroreversion may have been underestimated in this retrospective study. In patients without positive HBsAg, HBsAg and HBV DNA are usually performed when hepatitis has been suspected. However, our study demonstrates an increased risk not only for HBV reactivation but also for hepatitis flare; therefore, the conclusion of this study should be convincing. Third, the efficacy of long-term NA prophylaxis for kidney transplant patients with resolved HBV infection remains unclear. Although this study may stratify HBV-resolved patients in high risk of HBV reactivation, i.e., absence of anti-HBs and high-dose steroid maintenance, antiviral therapy prophylaxis cannot be directly recommended. A prospective study involving long-term NA prophylaxis versus periodic surveillance as controls would be valuable towards investigating both the risk of HBV reactivation and whether long-term NA prophylaxis could benefit liver and renal outcome.

In conclusion, the absence of baseline serum anti-HBs and the use of high-dose steroids may result in a higher risk of HBV reactivation in KTRs with resolved HBV infection, and the strategy of antiviral therapy prophylaxis may be defined according to the risk stratification for HBV reactivation.

## Data Availability

The original contributions presented in the study are included in the article/Supplementary Material, further inquiries can be directed to the corresponding authors.

## Abbreviations

ALT, alanine aminotransferase; anti-HBc, antibody to hepatitis B core antigen; anti-HBs, antibody to hepatitis B surface antigen; CI, confidence interval; DNA, deoxyribonucleic acid; HBeAg, hepatitis B e antigen; HBsAg, hepatitis B surface antigen; HBV, hepatitis B virus; HCV, hepatitis C virus; HLA, human leukocyte antigens; IQR, interquartile range; KTRs, kidney transplant recipients; MTP, methylprednisolone; NA, nucleos(t)ide analogue; SHR, subdistribution hazard ratio; ULN, upper limit of normal.

## References

[1] Global Observatory on Donation and Transplantation. International Report on Organ Donation and Transplantation Activities: Executive Summary 2019 (2021). Available at: http://www.transplant-observatory.org/wp-content/uploads/2021/04/glorep2019.pdf (Accessed April 24, 2022).

[2] Mathurin P Mouquet C Poynard T Sylla C Benalia H Fretz C Impact of Hepatitis B and C Virus on Kidney Transplantation Outcome. Hepatology (1999) 29:257–63. 10.1002/hep.510290123 9862875

[3] Yu TM Lin CC Shu KH Chuang YW Huang ST Chen CH Increased Risk of Hepatic Complications in Kidney Transplantation with Chronic Virus Hepatitis Infection: A Nationwide Population-Based Cohort Study. Sci Rep (2016) 6:21312. 10.1038/srep21312 26892933 PMC4759529

[4] Raimondo G Locarnini S Pollicino T Levrero M Zoulim F Lok AS Update of the Statements on Biology and Clinical Impact of Occult Hepatitis B Virus Infection. J Hepatol (2019) 71:397–408. 10.1016/j.jhep.2019.03.034 31004683

[5] Loomba R Liang TJ. Hepatitis B Reactivation Associated with Immune Suppressive and Biological Modifier Therapies: Current Concepts, Management Strategies, and Future Directions. Gastroenterology (2017) 152:1297–309. 10.1053/j.gastro.2017.02.009 28219691 PMC5501983

[6] Wong GL Wong VW Yuen BW Tse YK Yip TC Luk HW Risk of Hepatitis B Surface Antigen Seroreversion after Corticosteroid Treatment in Patients with Previous Hepatitis B Virus Exposure. J Hepatol (2020) 72:57–66. 10.1016/j.jhep.2019.08.023 31499132

[7] Chen MH Chen MH Chou CT Hou MC Tsai CY Huang YH. Low but Long-Lasting Risk of Reversal of Seroconversion in Patients with Rheumatoid Arthritis Receiving Immunosuppressive Therapy. Clin Gastroenterol Hepatol (2020) 18:2573–81.e1. 10.1016/j.cgh.2020.03.039 32205219

[8] Huang YH Hsiao LT Hong YC Chiou TJ Yu YB Gau JP Randomized Controlled Trial of Entecavir Prophylaxis for Rituximab-Associated Hepatitis B Virus Reactivation in Patients with Lymphoma and Resolved Hepatitis B. J Clin Oncol (2013) 31:2765–72. 10.1200/JCO.2012.48.5938 23775967

[9] Fabrizi F Martin P Dixit V Kanwal F Dulai G. HBsAg Seropositive Status and Survival after Renal Transplantation: Meta-Analysis of Observational Studies. Am J Transpl (2005) 5:2913–21. 10.1111/j.1600-6143.2005.01113.x 16303005

[10] Terrault NA Lok ASF McMahon BJ Chang KM Hwang JP Jonas MM Update on Prevention, Diagnosis, and Treatment of Chronic Hepatitis B: AASLD 2018 Hepatitis B Guidance. Hepatology (2018) 67:1560–99. 10.1002/hep.29800 29405329 PMC5975958

[11] European Association for the Study of the Liver. EASL 2017 Clinical Practice Guidelines on the Management of Hepatitis B Virus Infection. J Hepatol (2017) 67:370–98. 10.1016/j.jhep.2017.03.021 28427875

[12] Kanaan N Kabamba B Marechal C Pirson Y Beguin C Goffin E Significant Rate of Hepatitis B Reactivation Following Kidney Transplantation in Patients with Resolved Infection. J Clin Virol (2012) 55:233–8. 10.1016/j.jcv.2012.07.015 22921412

[13] Kim J Chung SJ Sinn DH Lee KW Park JB Huh W Hepatitis B Reactivation after Kidney Transplantation in Hepatitis B Surface Antigen-Negative, Core Antibody-Positive Recipients. J Viral Hepat (2020) 27:739–46. 10.1111/jvh.13279 32057171

[14] Lee J Park JY Huh KH Kim BS Kim MS Kim SI Rituximab and Hepatitis B Reactivation in HBsAg-Negative/Anti-HBc-positive Kidney Transplant Recipients. Nephrol Dial Transpl (2017) 32:722–9. 10.1093/ndt/gfw455 28339910

[15] Mei T Noguchi H Hisadome Y Kaku K Nishiki T Okabe Y Hepatitis B Virus Reactivation in Kidney Transplant Patients with Resolved Hepatitis B Virus Infection: Risk Factors and the Safety and Efficacy of Preemptive Therapy. Transpl Infect Dis (2020) 22:e13234. 10.1111/tid.13234 31856328

[16] Masutani K Omoto K Okumi M Okabe Y Shimizu T Tsuruya K Incidence of Hepatitis B Viral Reactivation after Kidney Transplantation with Low-Dose Rituximab Administration. Transplantation (2018) 102:140–5. 10.1097/TP.0000000000001870 28665891

[17] Meng C Belino C Pereira L Pinho A Sampaio S Tavares I Reactivation of Hepatitis B Virus in Kidney Transplant Recipients with Previous Clinically Resolved Infection: A Single-center Experience. Nefrologia (Engl Ed) (2018) 38:545–50. 10.1016/j.nefro.2018.02.004 29709320

[18] Querido S Weigert A Adragao T Rodrigues L Jorge C Bruges M Risk of Hepatitis B Reactivation in Hepatitis B Surface Antigen Seronegative and Core Antibody Seropositive Kidney Transplant Recipients. Transpl Infect Dis (2019) 21:e13009. 10.1111/tid.13009 30295412

[19] Paul S Dickstein A Saxena A Terrin N Viveiros K Balk EM Role of Surface Antibody in Hepatitis B Reactivation in Patients with Resolved Infection and Hematologic Malignancy: A Meta-Analysis. Hepatology (2017) 66:379–88. 10.1002/hep.29082 28128861 PMC6485929

[20] Chen MH Wu CS Chen MH Tsai CY Lee FY Huang YH. High Risk of Viral Reactivation in Hepatitis B Patients with Systemic Lupus Erythematosus. Int J Mol Sci (2021) 22:9116. 10.3390/ijms22179116 34502025 PMC8430791

[21] Cooper JE. Evaluation and Treatment of Acute Rejection in Kidney Allografts. Clin J Am Soc Nephrol (2020) 15:430–8. 10.2215/CJN.11991019 32066593 PMC7057293

[22] Tsai SF Lin MH Hsu CC Wu MJ Wang IK Chen CH. Trends of Kidney Transplantation from the 2020 Annual Report on Kidney Disease in Taiwan. J Formos Med Assoc (2022) 121:S20–S29. 10.1016/j.jfma.2021.12.009 35067427

[23] Mager DE Lin SX Blum RA Lates CD Jusko WJ. Dose Equivalency Evaluation of Major Corticosteroids: Pharmacokinetics and Cell Trafficking and Cortisol Dynamics. J Clin Pharmacol (2003) 43:1216–27. 10.1177/0091270003258651 14551176

[24] Perrillo RP Gish R Falck-Ytter YT. American Gastroenterological Association Institute Technical Review on Prevention and Treatment of Hepatitis B Virus Reactivation during Immunosuppressive Drug Therapy. Gastroenterology (2015) 148:221–44.e3. 10.1053/j.gastro.2014.10.038 25447852

[25] Fine JP Gray RJ. A Proportional Hazards Model for the Subdistribution of a Competing Risk. J Am Stat Assoc (1999) 94:496–509. 10.1080/01621459.1999.10474144

[26] Gray B. The Cmprsk Package. The Comprehensive R Archive network. (2022) Available at: https://cran.r-project.org/web/packages/cmprsk/cmprsk.pdf (Accessed October 31, 2022).

[27] Stuck AE Minder CE Frey FJ. Risk of Infectious Complications in Patients Taking Glucocorticosteroids. Rev Infect Dis (1989) 11:954–63. 10.1093/clinids/11.6.954 2690289

[28] Lin YC Chen YJ Lee SW Lee TY Chen YH Huang WN Long-Term Safety in HBsAg-Negative, HBcAb-Positive Patients with Rheumatic Diseases Receiving Maintained Steroid Therapy after Pulse Therapy. J Clin Med (2021) 10:3296. 10.3390/jcm10153296 34362079 PMC8347429

[29] Im YR Jagdish R Leith D Kim JU Yoshida K Majid A Prevalence of Occult Hepatitis B Virus Infection in Adults: A Systematic Review and Meta-Analysis. Lancet Gastroenterol Hepatol (2022) 7:932–42. 10.1016/S2468-1253(22)00201-1 35961359 PMC9630161

