# Supplementary figures and images for: Risk Stratification for Hepatitis B Virus Reactivation in Kidney Transplant Recipients With Resolved HBV Infection
Source: Transpl Int. 2023 Apr 13;36:11122. doi: 10.3389/ti.2023.11122 (PMC10134034; doi:10.3389/ti.2023.11122)

**Supplementary Material**

**Figure S1.** The follow-up time for HBV reactivation


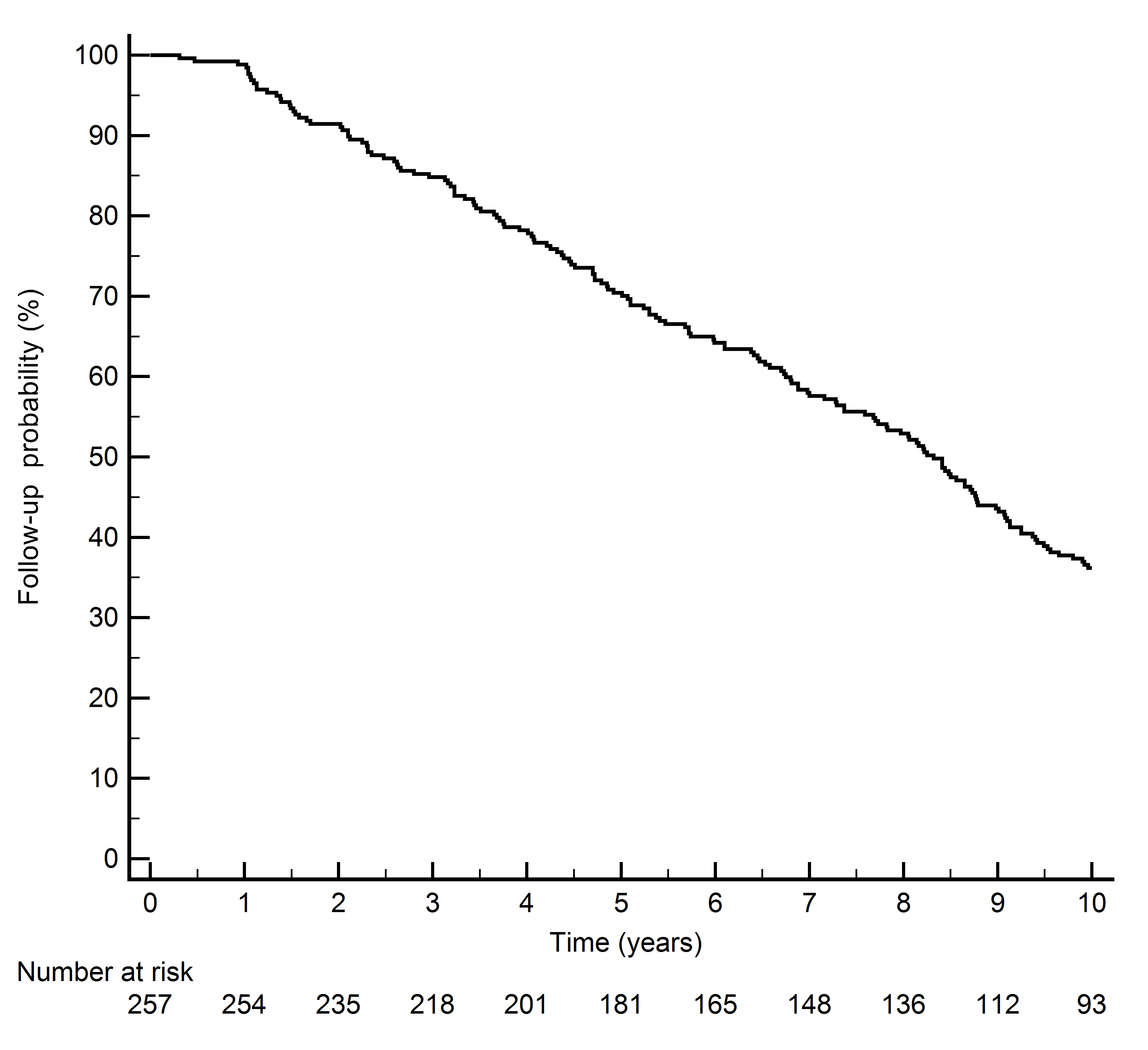

Supplement: Supplementary file 1 [file DataSheet1.docx]
